# A taxonomic, functional, and phylogenetic perspective on the community assembly of passerine birds along an elevational gradient in southwest China

**DOI:** 10.1002/ece3.3910

**Published:** 2018-02-06

**Authors:** Xuelian He, Kang Luo, Calum Brown, Luxiang Lin

**Affiliations:** ^1^ Key Laboratory of Tropical Forest Ecology Xishuangbanna Tropical Botanical Garden Chinese Academy of Sciences Kunming China; ^2^ University of Chinese Academy of Sciences Beijing China; ^3^ Karlsruhe Institute of Technology Institute of Meteorology and Climate Research Atmospheric Environmental Research (IMK‐IFU) Garmisch‐Partenkirchen Germany; ^4^ Southeast Asia Biodiversity Research Institute Chinese Academy of Sciences Yunnan China

**Keywords:** biodiversity, breeding season vs. wintering season, community assembly, elevational gradient, passerine birds

## Abstract

Integrating multiple facets of biodiversity to describe spatial and temporal distribution patterns is one way of revealing the mechanisms driving community assembly. We assessed the species, functional, and phylogenetic composition and structure of passerine bird communities along an elevational gradient both in wintering and breeding seasons in the Ailao Mountains, southwest China, in order to identify the dominant ecological processes structuring the communities and how these processes change with elevation and season. Our research confirms that the highest taxonomic diversity, and distinct community composition, was found in the moist evergreen broadleaf forest at high elevation in both seasons. Environmental filtering was the dominant force at high elevations with relatively cold and wet climatic conditions, while the observed value of mean pairwise functional and phylogenetic distances of low elevation was constantly higher than expectation in two seasons, suggested interspecific competition could play the key role at low elevations, perhaps because of relative rich resource result from complex vegetation structure and human‐induced disturbance. Across all elevations, there was a trend of decreasing intensity of environmental filtering whereas increasing interspecific competition from wintering season to breeding season. This was likely due to the increased resource availability but reproduction‐associated competition in the summer months. In general, there is a clear justification for conservation efforts to protect entire elevational gradients in the Ailao Mountains, given the distinct taxonomic, functional, and phylogenetic compositions and also elevational migration pattern in passerine bird communities.

## 1 INTRODUCTION

Identifying the relative importance of different ecological processes in controlling biodiversity and community composition across spatiotemporal scales is a fundamental objective of ecology (Sutherland et al., 2013). Particularly valuable, but challenging, are attempts to untangle the effects of abiotic and biotic filters (e.g., Chase, 2003; HilleRisLambers, Adler, Harpole, Levine, & Mayfield, 2012). Environmental filtering leads species with similar functional traits to occupy similar environmental niches, while interspecific competition may result in trait less similar between coexistence species (limiting similarity; Ackerly, Schwilk, & Webb, 2006; Lavorel & Garnier, 2002; MacArthur & Levins, 1967). If functional traits tend to be phylogenetically conserved, phylogenetic clustering (more similar compared to expectation) could be a result of environmental filtering while overdispersed phylogenetic structure suggests an effect of competitive exclusion. An absence of functional and phylogenetic structuring may suggest neutral process which highlights the relative importance of dispersal limitation and stochastic demography, but not the importance of the ecological or evolutionary differentiation between species (Hubbell, 2001; Webb, Ackerly, McPeek, & Donoghue, 2002).

Taxonomic, functional, and phylogenetic distribution patterns provide complementary approaches to detecting and untangling the mechanisms responsible for community assembly and are widely used for this purpose (e.g., Cadotte, Albert, & Walker, 2013; Huang, Stephens, & Gittleman, 2012; Monnet et al., 2014; Webb et al., 2002). However, few studies have so far integrated all three facets of biodiversity (but see e.g., Corbelli et al., 2015; Devictor et al., 2010; Monnet et al., 2014). Bird community assembly studies based on functional or/and phylogenetic aspect have tended to pay close attention to some specific bird families with high species diversity, such as Trochilidae, Thamnophilidae, and Parulidae (e.g., Gómez, Bravo, Brumfield, Tello, & Cadena, 2010; Graham, Parra, Rahbek, & McGuire, 2009; Lovette & Hochachka, 2006). In addition, some studies focus on a particular feeding guild or even all bird species coexistence (Dehling et al., 2014; Gianuca, Dias, Debastiani, & Duarte, 2014). Nevertheless, community assembly mechanisms are of particular interest between species with similar resource requirements and ecosystem roles that may interact intensely. As a big group of bird species coexist in forest (125 of 139 species in this research), passerine (Aves: Passeriformes) bird community is an ideal example to study community assembly mechanisms.

Mountain areas are also very suitable for the study of community assembly processes with striking elevational gradient of temperature and precipitation. Patterns in species diversity along elevational gradients are strong and relatively easy to measure, while may differ within and between taxonomic groups and climatic areas. Although a growing number of ecological and evolutionary hypotheses provide possible explanations for these differences, the underlying processes remain poorly understood (McCain, 2005, 2009; Sanders & Rahbek, 2012). Previous research has found that tropical bird communities at low elevations are functionally and/or phylogenetically more diverse than predicted by null models, whereas communities at high elevations are functionally and/or phylogenetically more similar than predicted by null models (hummingbird: Graham et al., 2009; frugivorous birds: Dehling et al., 2014, with species composition all retrieved from literature survey and abundance unweighted). The combination of taxonomic, functional, and phylogenetical analyses is particularly promising here, supplementing existing knowledge about community assembly processes.

Here, we describe the diversity patterns of passerine communities along an elevational gradient in the northern Ailao Mountains in southwest China, which is a part of the Indo‐Burma biodiversity hotspot and one of the Important Bird and Biodiversity Areas (IBAs) defined by BirdLife International (BirdLife International, 2016; Myers, Mittermeier, Mittermeier, da Fonseca, & Kent, 2000). The Mountain range shows an obvious vertical structuring in climate, with a cold and wet climate all year round at the high elevation and relative warm and dry in the low elevation (Wang, Tang, & Gao, 1988). The characteristic of seasonal vertical migration of bird community of the Ailao Mountains was thought to the reaction to climate and available resource change (Wang, 1986; Wang, Carpenter, & Young, 2000). We investigate the dominant processes structuring passerine bird communities, and how these processes change from wintering to breeding seasons, utilizing taxonomic, functional, and phylogenetic perspectives. Specifically, we test the following hypotheses: (1) that the functional and phylogenetic structure of the passerine bird community at high elevation is clustered, and contains the highest taxonomic diversity (Wang, 1986; Wang et al., 2000); (2) that environmental filtering is the driving force for passerine bird community assembly at high elevations, probably due to the prevailing cold and wet conditions, especially in the winter season; (3) On the contrary, interspecific competition is the driving force for community assembly at low elevations, which could be a result of richer resources due to warmer climate; (4) that more reproduction‐based resources need in the breeding season will increase interspecific competition across the whole elevational gradient, leading to decreases in the intensity of environmental filtering or even dominance of neutral or interspecific competition.

## 2 METHODS

### 2.1 Study area

The Ailao Mountains run through central Yunnan Province, China (E 100°54′–101° 30′, N 23°44′–24°44′), with vegetation following a typical midsubtropical mountain region vertical distribution (Liu, Wang, Lu, & Zheng, 1988; Wang et al., 2000). From the elevation of 2200 m to 2800 m, a large remnant of primary evergreen broadleaf forest exists (over 34,000 ha), protected by the Ailaoshan National Nature Reserve (Pang et al., 1988). Vegetation at lower elevations (below 2200 m) is dominated by coniferous forest, savanna shrub and monsoon evergreen broadleaf forests in inaccessible areas such as ravines (Pang et al., 1988). The latest estimate of the number of bird species in the Ailao Mountains is 462 (Wu et al., 2015). Our study was conducted in three forest plots (40 ha each) along an elevational gradient on the west slope of the northern Ailao Mountains (Figures S1 and S2). The high‐elevation Xujiaba plot (2,470 m, 24°32′N, 101°01′E) is moist evergreen broadleaf forest, with rich liana species and dominant species of *Lithocarpus xylocarpus*,* Castanopsis wattii, Schima sinensis,* and *Vaccinium mandarinorum* in high canopy density tree layer and *Fargesia spathacea* in shrub layer. Mixed coniferous and monsoon evergreen broadleaf forest is mostly distributed from elevation of 1300 m to 2000 m, the 40 ha Pizhang forest plot (1,602 m, 24°27′N, 100°58′E) is dominated by *Pinus kesiya var. langbianensis, Schima wallichii,* and *Engelhardtia roxburghiana* in tree layer and *Lyonia ovalifolia* and *Glochidion hirsutum* in shrub layer, the plot holds obvious forest stratification and developed shrub layer and herb layer (Pang et al., 1988). The low‐elevation Daduanyao plot (1,270 m, 24°26′N, 100°53′E) is a mixed forest of *Pinus kesiya var. langbianensis* and savanna shrub, with sparse tree layer and thick shrub and herb layer dominated by *Woodfordia fruticosa* and *Heteropogon contortus,* respectively.

### 2.2 Bird surveys

According to Wang (1986), the wintering bird community composition of Ailao Mountains became stable in December and the breeding season from April to June. In this study, the bird surveys were conducted during the two seasons of the bird life cycle: wintering and breeding, specifically, from 12th December 2014 to 4th February 2015 and from 23rd June to 27th August 2015. Three no straight transects with randomly selected start points and direction were chosen in each 40 ha forest plot, with a minimum distance of 200 m from each other. In total, the length of the transects was 2,520 m, 2,060 m, and 2,230 m for Xujiaba, Pizhang, and Daduanyao plot, in addition, the survey transect was the same in both seasons for each plot. Two experienced observers (Xuelian He and Kang Luo) walked along the transects at a speed of 1 km/h, and one observer recorded all birds heard and observed with binoculars. The surveys were carried out from 30 min after sunrise to 11:00 in the morning and from 15:30 to 30 min before sunset in the afternoon under clear weather conditions, discarding foggy and windy days. Surveys were repeated eight times (a morning and afternoon survey was counted as one repeat) for each transect and the first and the last repeat for each transect kept a time interval of 2 months to insure the complete of the species detected (Ralph, Geupel, Pyle, Martin, & DeSante, 1993). The detection distance was not recorded in the surveys, and we then used the maximum number of individuals detected by observers within one of the eight repeats as the best estimator for one transect, resulting in a composite estimate of species’ relative abundances for one plot in certain season (Jankowski & Rabenold, 2007). The bird taxonomy and nomenclature in our study follows the BirdLife taxonomic checklist v8.0 (BirdLife International, 2015).

### 2.3 Trait data

Six types of mostly used bird functional traits related to resource utilization and life history strategy were selected in this study: two continuous traits (body mass and generation length), one binary trait (migratory status), and three categorical traits (diet, foraging method, and foraging location). The three categorical traits included two with five and one with four binary attributes which were not mutually exclusive (17 traits in total, Table S1, Ding, Feeley, Wang, Pakeman, & Ding, 2013; Flynn et al., 2009; Luck, Carter, & Smallbone, 2013; Newbold et al., 2013). Body mass data were mostly compiled from Dunning (2007), with supplementary data taken from del Hoyo, Elliott, Sargatal, Christie, and de Juana (2015) and Zhao (2001). Information about bird generation length (the average age of parents in the population) and migratory status were collected from BirdLife International's World Bird Database (available online at http://www.birdlife.org/datazone/home). There are 30 species with no generation length in the database which were assigned the value of the most closely related available species. No nomadic bird species were recorded in this study, altitudinal and full migrants were classified as migrant. Diet, foraging method, and foraging location data were mostly obtained from Yang and Yang (2004), which summarized 508 passerine bird species recorded in Yunnan Province before 2004. The Eco‐biological Characteristics section gathered the habitat, foraging information, and breeding ecology for each species. All the descriptive texts about diet, foraging method, and foraging location were translated to binary attributes (1 and 0). In detail, we treat “mostly” as 1 and “occasionally”, “a bit” as 0, if the description mentioned “and”, both the two trait will be assigned to 1. Specific definition of the four foraging methods was as follows: (1) glean: to pick static or slowly moved food item from a nearby substrate a surface such as a tree, branch, grass, or leaves; (2) probe: to insert the bill into cracks or holes in firm substrate or directly into softer substrates such as moss or mud to capture hidden food; (3) sally: to fly from a perch to attack a food item in the air but returning to a perch to feed; (4) leap: snatching food, usually insects, with the bill while in flight and consuming it without perching. Foraging method and location information were supplementally retrieved from Zhao (2001) and bird survey record for some species (shown in Result section).

### 2.4 Phylogeny and phylogenetic signal

The phylogenetic trees used in this study were derived from the first mega phylogeny of 9993 extant birds species constructed by Jetz, Thomas, Joy, Hartmann, and Mooers (2012), who combined relaxed clock molecular trees of well‐supported avian clades with a fossil‐calibrated backbone with representatives from each clade (see details in reference Jetz et al., 2012). The Jetz tree represents the most comprehensive and latest phylogenetic information for extant birds and can be applied to species‐level inference in spite of remaining uncertainty (Barnagaud et al., 2014; Mayr, 2013). A total of 1000 “stage 2” trees were subsampled, then a majority‐rule rooted consensus tree of the 1,000 trees was built using Mesquite 3.10 (Figure S3, Maddison & Maddison, 2016). We demonstrate the results from the “Ericson” model tree in this paper as both the “Ericson” and “Hackett” trees showed similar phylogenetic diversity and structure in our data.

In order to assess the extent to which the phylogenetic patterns of the passerine bird community mirrored the patterns of selected functional traits in our study, the phylogenetic signal of each of the 17 traits was measured (Losos, 2008). For the continuous traits body mass and generation length, the K‐statistic was used to quantify the phylogenetic signal (Blomberg, Garland, & Ives, 2003). Values of K exceeding one generally indicate a strong phylogenetic signal in the trait data while values below one indicate a weak phylogenetic signal. For the 15 binary traits, the phylogenetic signal was calculated using the D‐statistic (Fritz & Purvis, 2010). The D‐statistic approaches 0 as the trait phylogenetic signal increases; negative values of the D‐statistic indicate that the binary trait is more conserved than expected under Brownian motion (Corbelli et al., 2015; Fritz & Purvis, 2010). The K‐statistic and D‐statistic were calculated in the R packages “phytool” and “caper”, respectively (Blomberg et al., 2003; Fritz & Purvis, 2010; R Development Core Team, 2015).

### 2.5 Data analyses

Bird species diversity and evenness were estimated by the species richness (SR) and Pielou's evenness index, respectively, in the R package “vegan” (Oksanen, 2015). To visualize the taxonomic composition of the passerine bird communities in different seasons along the elevational gradient, we carried out nonmetric multidimensional scaling (NMDS) on Bray–Curtis distances using the “metaMDS” function in “vegan”. The relative abundances of bird species were square root transformed and then submitted to Wisconsin double standardization as recommended for NMDS analysis (Oksanen, 2015). Season and elevation were fitted to ordination using the “envfit” function in “vegan”.

Functional diversity was measured as functional richness (FRic), which calculates the volume of the functional space occupied by the community (Cornwell, Schwilk, & Ackerly, 2006; Villéger, Mason, & Mouillot, 2008). A community with high functional richness has coexisting species that occupy a large functional volume (space) or includes species with distinct functional traits on the margins of the volume (Cornwell et al., 2006). FRic was calculated using the function “dbFD” in the R package “FD” (Laliberté, Legendre, & Shipley, 2014). Faith's PD (phylogenetic diversity) was used to measure phylogenetic diversity in our study (Faith, 1992). Calculating the minimum total length of all the phylogenetic branches required to span a given set of taxa on the phylogenetic tree, larger Faith's PD values can be expected to correspond to greater expected feature diversity. The R package “picante” was used to calculate Faith's PD (Kembel et al., 2010).

A standardized effect size of the mean pairwise functional distance (S.E.S. PW) was used to quantify the functional structure of the bird communities. Functional distances between all individuals within a local community were calculated using the function “gowdis” in the R package “FD”, which computes the Gower dissimilarity from different trait types (continuous, ordinal, nominal, or binary; Laliberté et al., 2014). The S.E.S. PW was calculated as follows:

$$\text{S.E.S. PW}\;=\;-1\;\times \;\left({\text{PW}}_{\mathrm{obs}}\;-\;\text{Mean}\left({\text{PW}}_{\mathrm{null}}\right)\right)/\text{SD}\left({\text{PW}}_{\mathrm{null}}\right)$$

where PW_obs_ is the observed value of mean pairwise functional distances between all individuals within a local community, PW_null_ is the mean value from a null distribution where species names were randomly shuffled on the tips of the community traits dendrogram 999 times, and the SD (PW_null_) is the standard deviation of the null distribution.

The phylogenetic structure was determined using the net relatedness index (NRI), which is the standardized effect size for the mean pairwise phylogenetic distance (MPD) of all species in the local community (Webb et al., 2002). With the same null model as that used in the functional calculation for phylogenetic structure, the species names on the tips of the phylogenetic tree were randomly shuffled, and the NRI was calculated as follows:

$$\text{NRI}\;=\;-1\;\times \;\left({\text{MPD}}_{\mathrm{obs}}\;-\;\text{Mean}\left({\text{MPD}}_{\mathrm{null}}\right)\right)/\text{SD}\left({\text{MPD}}_{\mathrm{null}}\right)$$

A negative value of S.E.S.PW or NRI indicates that a community is functionally/phylogenetically overdispersed based on limiting similarity (MacArthur & Levins, 1967), whereas a positive value indicates functional/phylogenetic clustering (more similar; Cavender‐Bares, Kozak, Paul, Fine, & Kembel, 2009; Webb et al., 2002). Functional and phylogenetic structure analyses were implemented in the R package “picante” with “taxa.labels” null model (Kembel et al., 2010; Swenson, 2014). One‐sample *t* test was used to determine whether S.E.S.PW and NRI of each community were significantly different from zero (Edwards et al., 2014). All the indices of the bird community in this study were analyzed separately by wintering and breeding seasons and weighted by species abundance.

## 3 RESULTS

**Figure 1:**
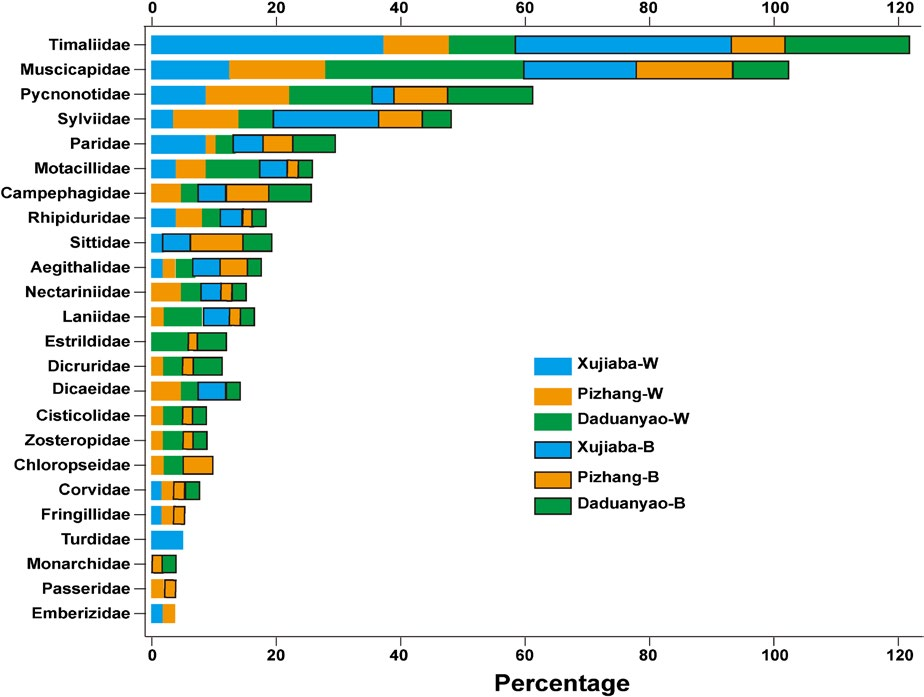
Percentage of species belongs to 24 families of the six passerine bird communities in Ailao Mountains. The community names with a ‐W and ‐B appended represented wintering and breeding season, respectively

**Figure 2:**
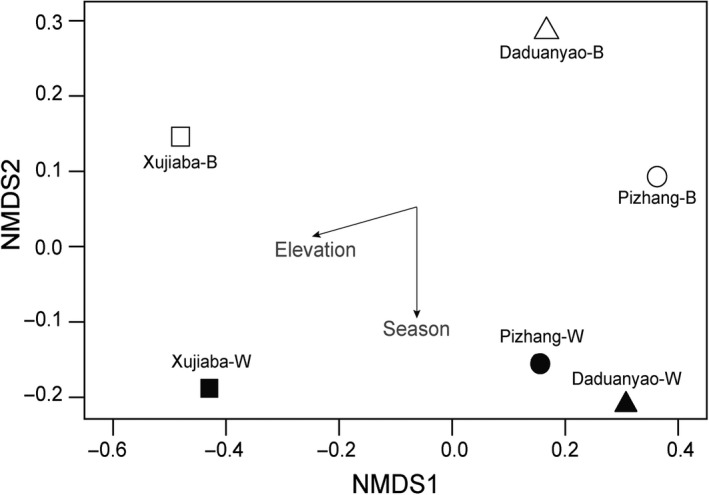
Ordination plots of nonmetric multidimensional scaling (NMDS) for the passerine communities (stress = 0). Solid black square, triangle, and circle represent winter season passerine communities, whereas the open ones show breeding season communities. NMDS was based on Bray–Curtis distances of species abundances of each community

A total of 139 bird species (3,125 detections) were recorded in the three plots in the two seasons, including 125 passerine species and 14 nonpasserine species belong to Galliformes, Columbiformes, Cuculiformes, Accipitriformes, Piciformes, Columbiformes, and Psittaciformes (Table S2). The species accumulation curve (SAC) showed eight repeats were sufficient to record the passerine bird community of the three 40 ha forest plots both in wintering and breeding seasons (Figure S4). With the similar transect length of the three forest plot and the same in different seasons, we believe the species relative abundance result from the eight repeats in this study is reliable even the vegetation structure of the three forest plot was not the same. The passerine bird community of the high elevation had the highest species richness, but the lowest species evenness observed mean pairwise functional and phylogenetic distance in both seasons compared to the other community (Table 1). More than 35% of passerine species of the high‐elevation Xujiaba community belong to Timaliidae (39% for wintering and 37% for breeding season), and only 13 families of passerine birds were recorded (20 and 21 for Pizhang and 17 and 19 for Daduanyao in two seasons specifically, Figure 1). The community composition of the high elevation could be separated along NMDS1 from the other two elevations (Figure 2). Twenty‐nine passerine species inhabited the same elevation in both seasons and only three species *Aegithalos concinnus, Pomatorhinus ruficollis,* and *Motacilla alba* existed in all elevations in both seasons. Seasonal vertical migration along elevations was recorded: nine species moved to lower elevations in the breeding season and four species only found at one or two elevations in the wintering season expanded to all elevations in the breeding season.

**Table 1:** Five indices of passerine bird communities of the three forest plots in two seasons of Ailao Mountains

Seasons	Plots	SR	SE	FRic	PD	PW	MPD
Wintering	Xujiaba	**46**	0.91	**227.74**	903.27	0.30	56.04
	Pizhang	44	**0.93**	317.05	**1056.08**	0.33	67.47
	Daduanyao	35	**0.93**	154.28	784.56	**0.34**	**67.79**
Breeding	Xujiaba	**57**	0.86	230.95	**1099.59**	0.28	55.18
	Pizhang	43	**0.88**	**448.36**	1004.62	**0.32**	**70.86**
	Daduanyao	43	0.87	395.56	997.79	0.31	69.80

SR—species richness; SE—species eveness (Pielou's index); FRic—functional richness; PD—Faith's phylogenetic diversity; PW—observed value of mean pairwise functional distances; MPD—observed value of mean pairwise phylogenetic distance. The largest values among the three passerine communities in each season were shown in bold.

Functional traits data of the 125 passerine bird species observed are listed in Supplementary Material Table S3. The *K* values of body mass and generation length were close to 1, with a *p* value of.001, demonstrating that these two functional traits hold significant phylogenetic signal (Table 2). *D* statistics revealed weak phylogenetic signals in migratory status and understory foraging (0 < *D*_obs_ < 1) but strong signals for the rest of the 13 binary traits (*D*_obs_ < 0). Under a significance level of 0.01, all of the binary traits differed significantly from random distributions along the phylogeny, but not from the Brownian distribution model (Table 1).

**Table 2:** Phylogenetic conservatism tests for 17 Passeriformes bird functional traits of Ailao Mountains. *p* (*D* < 1) is the significance level in the test of random distribution of traits along phylogeny, and *p* (*D* > 0) is the result of testing whether *D* is significantly different from zero

Traits	Continuous	
	*K*	*p*
Body mass	0.792	.001
Generation length	1.079	.001

	Binary		
	*D*	*p* (*D < 1*)	*p* (*D > 0*)
Migratory status	0.518	.002	.068
Seeds	−0.03	0	.56
Nectar	−1.414	0	.949
Fleshy fruits	−0.348	0	.828
Invertebrates	−0.249	0	.881
Vertebrate	−1.438	.005	.893
Glean	−0.392	0	.873
Probe	−0.063	0	.576
Sally	−0.447	0	.878
Leap	−0.69	0	.952
Water	−1.199	0	.946
Ground	−0.527	0	.916
Understory	0.028	0	.492
Midstorey	−0.497	0	.917
Canopy	−0.282	0	.799

**Figure 3:**
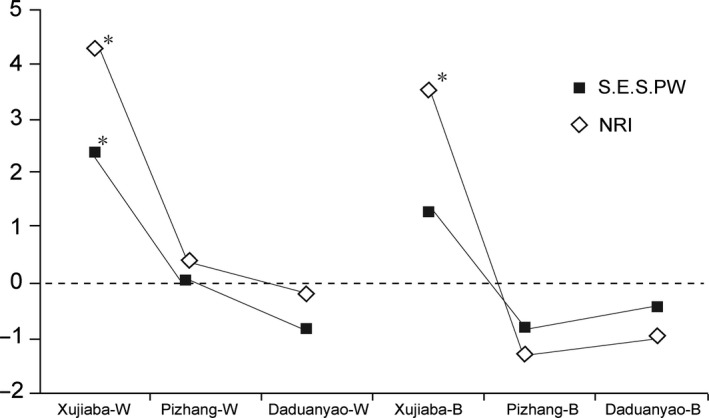
Functional and phylogenetic structure of passerine bird communities of three plots of Ailao Mountains in wintering (‐W) and breeding (‐B) seasons. S.E.S. PW—standardized effect size of mean pairwise functional distance, NRI—net relatedness index. Asterisks denote significant clustering or overdispersion compared to the randomizations (*p *<* *.01)

The mean pairwise functional and phylogenetic distance of passerine communities from high‐elevation plot were significantly lower (*p *<* *.01) than the null distribution for both seasons (except the S.E.S. PW for breeding season, *p *=* *.201); meanwhile, it was not significantly higher than the null distribution for low‐elevation plots. For the middle‐elevation plot, the mean pairwise functional and phylogenetic distances were not significantly lower (nearly the same) and higher than the null distribution in the wintering season and breeding season, respectively (Figure 3). The S.E.S.PW and NRI for all communities hold a Pearson's correlation of 0.95 (*p *=* *.004). Paired sample *t* test showed the S.E.S.PWs between wintering season and breeding season were not significantly different (*p *=* *.494), but the NRIs of breeding season were significantly lower than wintering season (*p *=* *.027).

## 4 DISCUSSION

This study provides a detailed description of the taxonomic, functional, and phylogenetic structure of extratropical passerine bird communities in two life cycle seasons along an elevational gradient in the Ailao Mountains. Our findings confirm the highest species richness and distinct composition of the protected moist evergreen broadleaf forests in two seasons and also highlight a tendency of interspecific competition as the driving force in shaping community structure of the passerine bird community from wintering season to breeding season. This research contributes to better knowledge about underling mechanisms and seasonal dynamics in subtropical passerine bird communities.

Ecologists have come to realize that phylogenies should be useful tools for predicting community structure, given phylogenetic conservatism of traits (Losos, 2008). In this study, functional and phylogenetic structure was significantly correlated in each passerine assemblage (Pearson's correlation, *p *=* *.004), with significant phylogenetic signals of all 17 traits. Early studies of animal communities along elevational gradients indicated clustered functional or/and phylogenetic structures at high elevations and dispersed structures at low elevations (e.g., bird: Graham et al., 2009; Dehling et al., 2014; ant: Machac, Janda, Dunn, & Sanders, 2011). Our research, some of the first to consider bird communities between two life cycle seasons in a subtropical region, suggests that these findings hold more generally than previously known.

The hypotheses 1 and 2 were supported. McCain (2009) summarized four general bird taxonomic diversity patterns in nearly equal frequency on mountains (Figure 1 in McCain, 2009). Although the bird diversity of mountaintop mossy dwarf forest was not surveyed in this study (2800–3000 m, <25 bird species were recorded in the breeding season according to the database of Chinese National Ecosystem Research Network: http://www.cnern.org.cn/index.jsp), our results demonstrated a right‐shifted McCain's “mid‐elevational peak” of bird taxonomic diversity of the Ailao Mountains, which confirms the highest taxonomic richness and the importance of the moist evergreen broadleaf forest, as previously suggested by other studies on both the west and east slope of the mountain range (Wang, Li, Fang, & Yang, 1998; Wang et al., 2000). With the same sampling effort, such species diversity patterns could be generally attributed to a clear difference in vegetation type along elevational gradients. The protected high‐elevation primary moist evergreen broadleaf forests hold a stable vegetation structure compared to the other mixed forest (mixed coniferous and monsoon evergreen broadleaf forest and mixed coniferous forest and savanna shrub for Pizhang and Daduanyao plot, respectively) in lower elevations, resulting in a concentrated and distinct taxonomic distribution in both seasons (more than 50% belonged to two families Timaliidae and Muscicapidae, Figure 1; Joshi, Bhatt, & Thapliyal, 2012; Lee & Rotenberry, 2005). Independent species radiations could produce the observed high taxonomic species richness but clustered functional and phylogenetic structure of the high elevational community (Emerson & Gillespie, 2008).

Distribution patterns of biotic communities along an elevational gradient could be affected by several physical and ecological factors, which can vary with altitude, climate, habitat structure, and resource availability (Lomolino, 2001). The Ailao Mountains show a characteristic of clear wet and dry seasons, with nearly 87% of precipitation occurring from May to October. In addition, the mountain range has an obvious vertical structure in climate, with the mountaintop having a mean annual temperature of 11.1°C compared to 18.1°C in the foothills, with precipitation 67% more than the foothills over the course of a year, resulting in a cold and wet climate all year round at high elevation (Wang, Tang, & Gao, 1988). The climate of the high‐elevation forest could therefore present direct metabolic challenges and indirect food resource limitations to certain bird species, favoring species adapted to cold and wet conditions, especially in wintering season (S.E.S.PW and NRI of passerine community of high elevation were significantly different from zero except to the S.E.S. PW in breeding season).

There was no sufficient evidence to back up the hypothesis 3, but the hypothesis 4 was partial proved in our study. The low‐elevation plots, conversely, have warmer and drier climates, combined with complex vegetation structure including increased canopy openness and a high‐density shrub layer, which provide more diverse opportunities for specialization for passerine species. The low‐elevation passerine community showed higher observed mean pairwise functional and phylogenetic distance, but not significantly different from expectation. The statistically null distribution could also be a result of human disturbance, for example, occasional domestic animal grazing and timber plantations (Wang et al., 2000; Wu, 1987). According to the paired sample *t* test, significant lower of NRIs give evidence of the strength of environmental filtering was reduced and interspecific competition was increased from wintering to breeding season. This pattern of structure could be a result of reproduction‐associated competition in the summer.

The fact that the high‐elevation plot sustained the highest species richness, but clustered functional and phylogenetic structure in both seasons underlines the need for taking account of multiple facets of biodiversity. On the other hand, anthropogenic climate change is now affecting many biological and ecological processes, from population distributions to community structure (Scheffers et al., 2016). Species would either adapt locally or to shift their range to track preferred climatic conditions could be result in disturbed species interactions and novel, potentially unstable community structures (Chen, Hill, Ohlemüller, Roy, & Thomas, 2011; Freeman & Freeman, 2014; Wittwer, O'Hara, Caplat, Hickler, & Smith, 2015). Taking the three facets of biodiversity into consideration along with vertical migration characteristics of passerine communities, we recommend conservation efforts span entire elevational gradients in the Ailao Mountains (Wang et al., 2000; Wu, Liu, Fang, Zhang, & Yang, 2016).

In conclusion, our research confirms highest species richness and distinct composition of the protected moist evergreen broadleaf forests in Ailao Mountains. We also highlight a constant clustered functional and phylogenetic structure for high elevations and an absence of functional and phylogenetic structure for low elevation, with a tendency for interspecific competition in the breeding season to shape passerine bird community structure. Bird community assembly research involving more elevational gradients in two life cycle seasons of extra tropical region is encouraged.

## CONFLICT OF INTERESTS

None declared.

## AUTHOR CONTRIBUTIONS

XH and LL conceived the research ideas; XH and KL collected data; XH and LL performed the analyses; XH, LL, and CB wrote the manuscript, which was commented on and improved by all the authors.
