# Supplementary material for: A taxonomic, functional, and phylogenetic perspective on the community assembly of passerine birds along an elevational gradient in southwest China
Source: Ecol Evol. 2018 Feb 6;8(5):2712–20. doi: 10.1002/ece3.3910 (PMC5838049; doi:10.1002/ece3.3910)
Supplement: Supplementary file 1 [file ECE3-8-2712-s001.docx]

**Supplementary material**
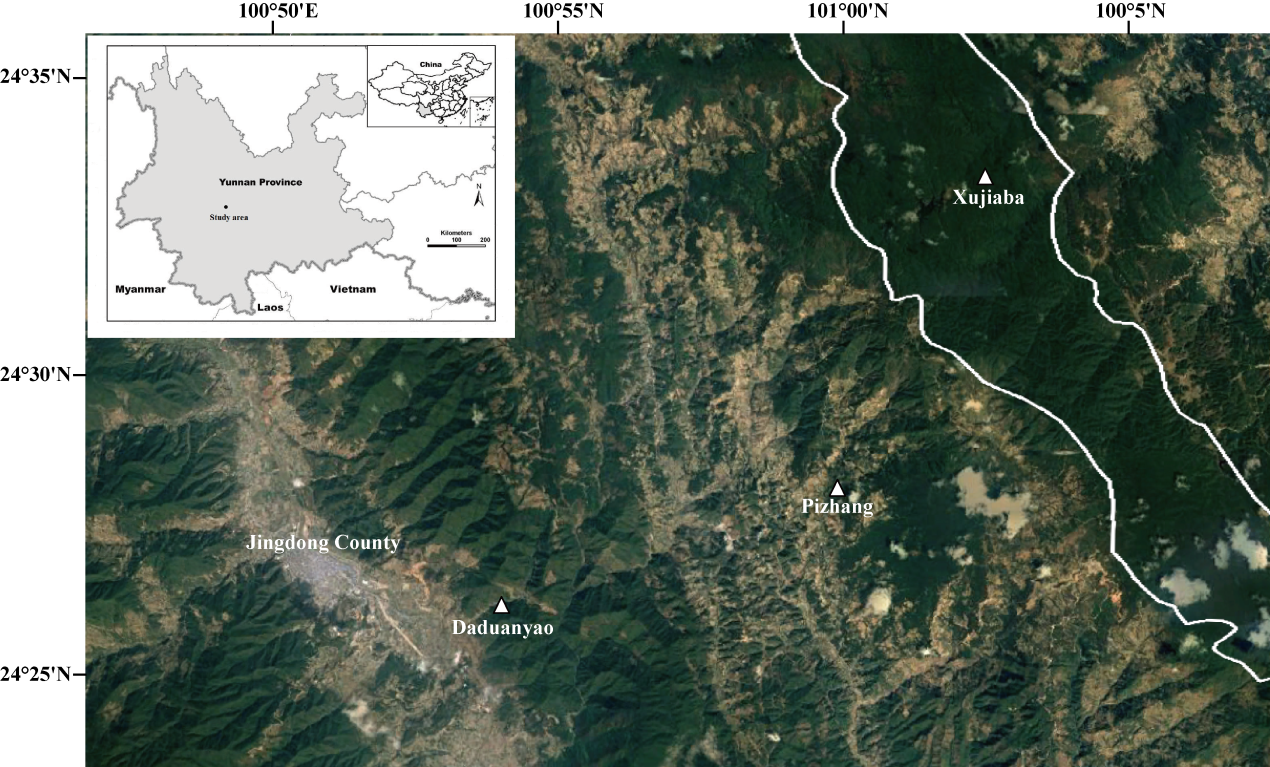


**FIGURE S1** Location of the three forest plots in the Ailao Mountains, Yunnan Province, China. The map of forest cover was obtained from Google Earth 7.1.2.2041 (http://www.google.com/earth/index.html), supplied by Data SIO, NOAA, U.S. Navy, NGA, GEBCO ©2016 Google, while the one on the top left corner was generated by ArcGis 10.1 (http:// www.esri.com) and base maps. The two maps were combined and edited by X. He using Adobe Illustrator CS5 (https://www.adobe.com/cn/products/catalog.html). The white line represented the boundary of the Ailaoshan National Nature Reserve.


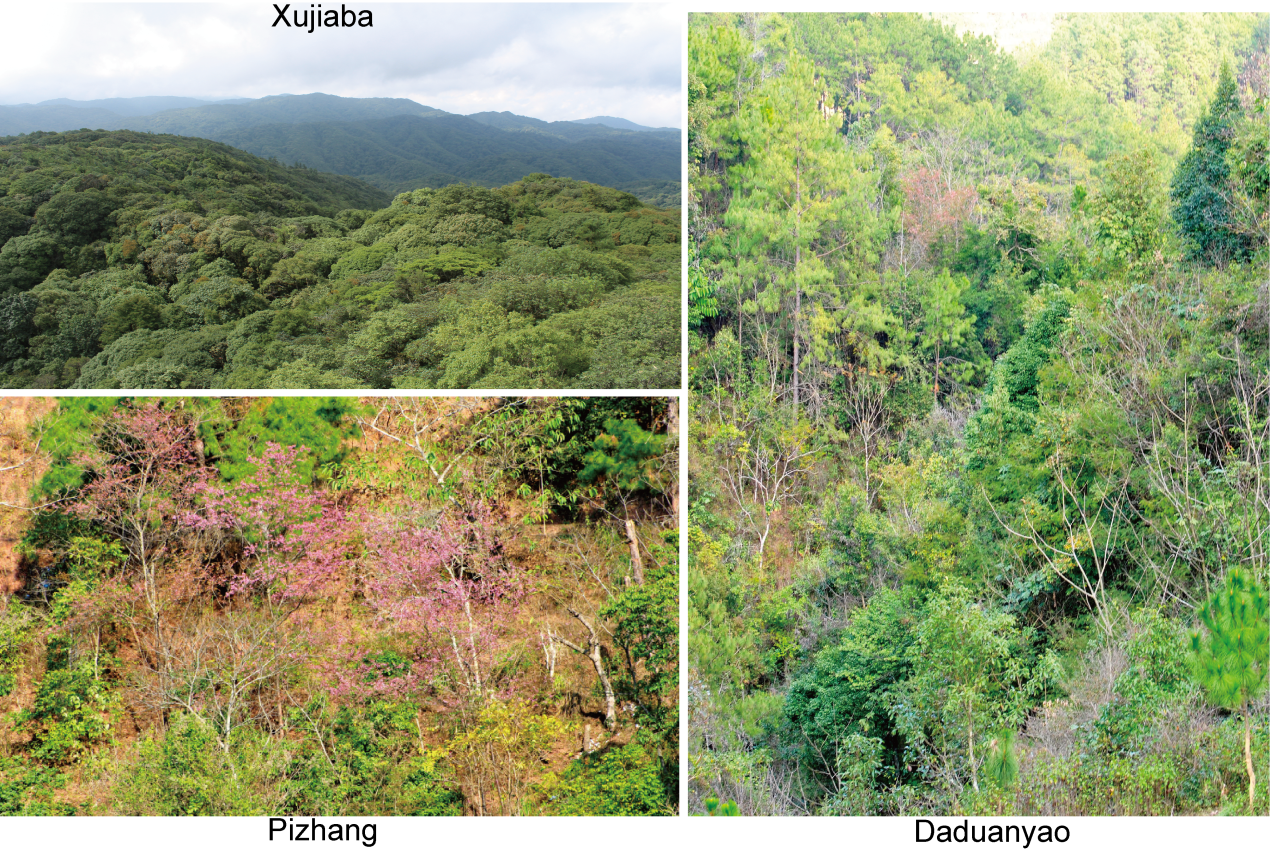


**FIGURE S2** Pictures of the three forest plots along an elevational gradient in this study (taken in summer season for Xujiaba by Kang Luo and winter season for Pizhang and Daduanyao by Xuelian He).

**
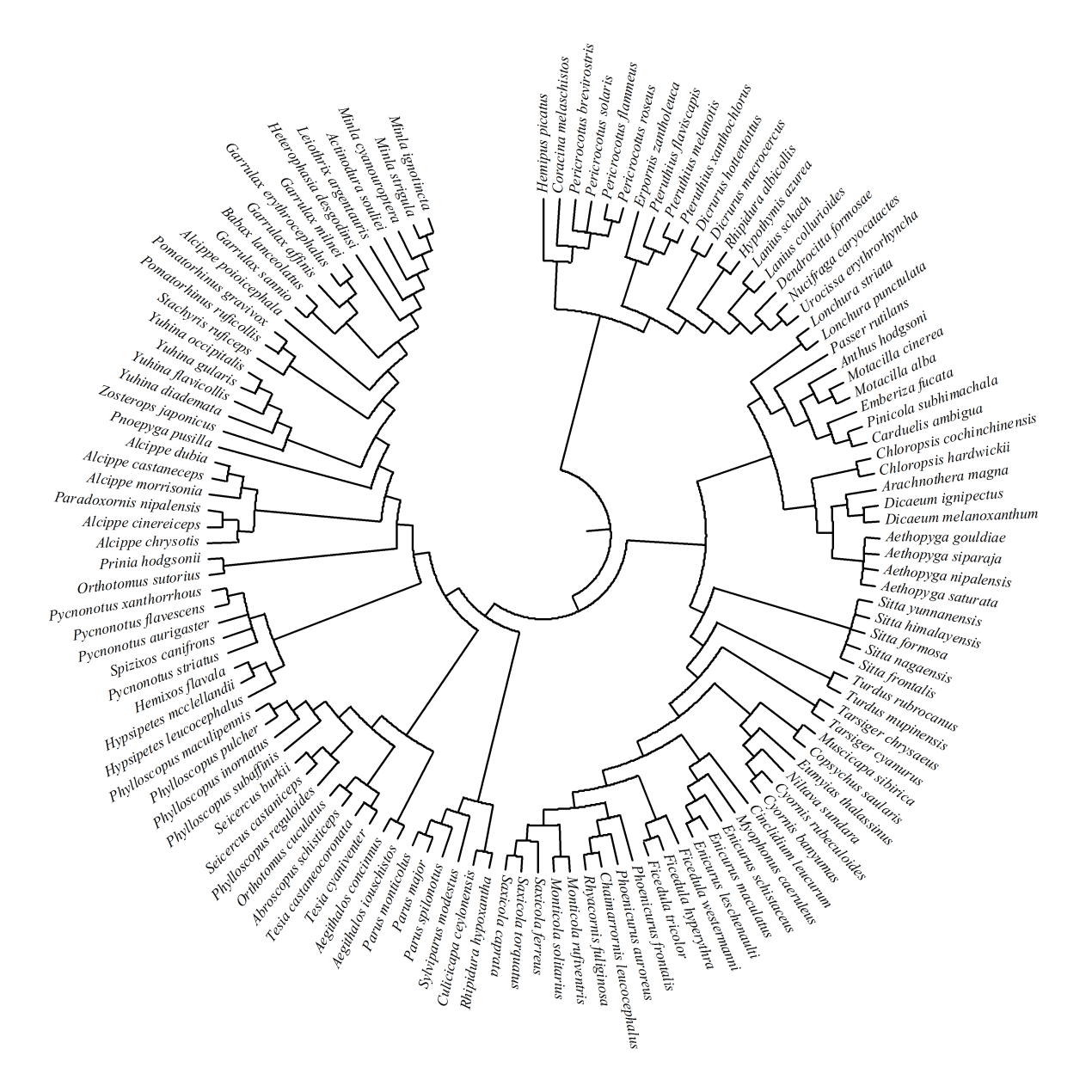
**

**FIGURE S3** Phylogenetic consensus tree of the 125 passerine birds in three plots of Ailao Mountains based on Jetz, Thomas, Joy, Hartmann, & Mooers (2012).


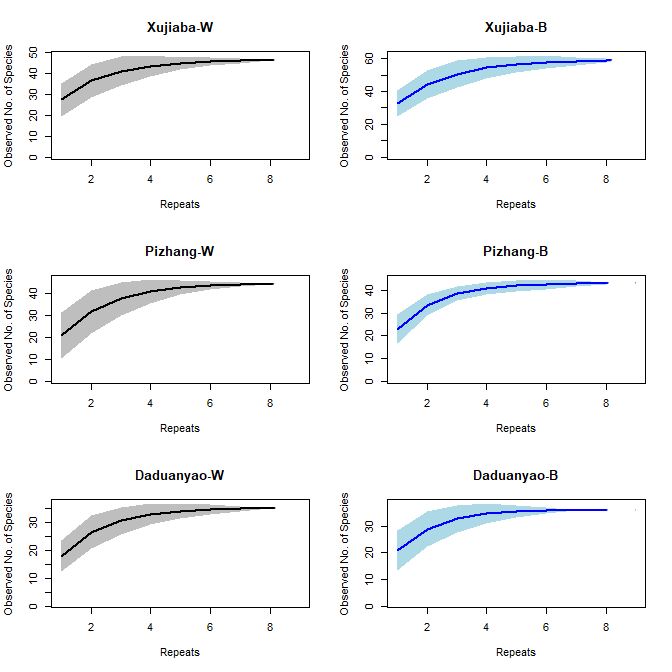


**FIGURE S4** Bird species accumulation curve (SAC, with exact method, using "specaccum" functiona in R package "vegan") of the three forest plots in two seasons of Ailao Mountains. The community names with a -W and -B appended represented wintering and breeding season.

**TABLE S1** Traits used to measure avian community functional diversity and structure.

| **Trait type** | **Trait** | **Description** | **Justification** |
| --- | --- | --- | --- |
| Resource quantity | Body mass | Average of male and female values weighted by sample size, continuous (g). | Relates to the resource and/or energy requirements, metabolism, and territory size (Ernest, et al., 2003; Speakman, 2005). |
| Life-history | Generation length | The average age of breeding individuals, continuous (yrs). | Tradeoff between survival and reproduction (Krüger, 2005). |

| Migration | Migratory status | Binary traits: non-migrants, migrants (nomadic, altitudinal migrants, or full migratory). | May impose seasonality on any ecosystem functions performed by migrating birds (Newbold, Butchart, Şekercioğlu, Purves,. & Scharlemann, 2012). |
| --- | --- | --- | --- |
| Diet | Seeds | Five categories traits, Binary. | A important functional effect trait of bird, e.g. seed dispersal, pollination and pest control(Greenberg et al., 2000; Van Bael, Brawn, & Robinson, 2003). |
|  | Nectar |  |  |
|  | Fleshy fruits |  |  |
|  | Invertebrates |  |  |
|  | Vertebrate |  |  |
| Foraging method | Glean | Four categories traits, Binary. | Foraging method and location are key foraging traits of bird related to the resource utilization (Holmes, Bonney, & Pacala, 1979; Sekercioglu [Daily](http://www.pnas.org/search?author1=Gretchen+C.+Daily&sortspec=date&submit=Submit), & [Paul Ehrlich](http://www.pnas.org/search?author1=Paul+R.+Ehrlich&sortspec=date&submit=Submit), 2004). |
|  | Probe |  |  |
|  | Sally |  |  |
|  | Leap |  |  |
| Foraging location | Water | Five categories traits, Binary. |  |
|  | Ground |  |  |
|  | Understory |  |  |
|  | Midstorey |  |  |
|  | Canopy |  |  |

**The "other plant material" (such as weed and plant roots) is also a selected diet trait in this study but only *Babax lanceolatus* get "1", so the trait was deleted in our following analyses and did not show in the Table.**

**References**

Ernest, S.K.M., Enquist, B.J., Brown, J.H., Charnov, E.L., Gillooly, J.F., Savage, V.M., ... Tiffney, B. (2003) Thermodynamic and metabolic effects on the scaling of production and population energy use. *Ecology Letters*, 6, 990-995.

Speakman, J.R. (2005) Body size, energy metabolism and lifespan. *Journal of Experimental Biology*, 208, 1717-1730.

Krüger, O. (2005) Age at first breeding and fitness in Goshawk *Accipiter gentilis*. *Journal of Animal Ecology*, 74, 266-273.

Greenberg, R., Bichier, P., Cruz Agnon, A., MacVean, C., Perez, R. & Cano, E. (2000) The impact of avian insectivory on arthropods and leaf damage in some Guatemalan coffee plantations. *Ecology*, 81, 1750-1755.

Newbold, T., Butchart, S.H.M., Şekercioğlu, Ç.H., Purves, D.W. & Scharlemann, J.P.W. (2012) Mapping functional traits: comparing abundance and presence-absence estimates at large spatial scales. *PLoS one*, 7, e44019.

Van Bael, S.A., Brawn, J.D. & Robinson, S.K. (2003) Birds defend trees from herbivores in a Neotropical forest canopy. *Proceedings of the National Academy of Sciences USA*, 100, 8304-8307.

Holmes, R.T., Bonney, R.E.Jr & Pacala, S.W. (1979) Guild structure of the Hubbard Brook bird community: a multivariate approach. *Ecology*, 60, 512-520.

Şekercioğlu, Ç. H., [Daily](http://www.pnas.org/search?author1=Gretchen+C.+Daily&sortspec=date&submit=Submit) G.C., & [Ehrlich](http://www.pnas.org/search?author1=Paul+R.+Ehrlich&sortspec=date&submit=Submit) P.R. (2004). Ecosystem consequences of bird declines. *Proceedings of the National Academy of Sciences USA*, 101, 18042-18047.

**TABLE S2** Plots × species abundance matrix of the 125 passerine birds in the wintering and breeding seasons of three elevational gradient plots on Ailao Mountains.

|  | **XJB-W** | | **PZ-W** | | **DDY-W** | | | **XJB-B** | | | **PZ-B** | | **DDY-B** |
| --- | --- | --- | --- | --- | --- | --- | --- | --- | --- | --- | --- | --- | --- |
| *Coracina melaschistos* | 0 | | 2 | | 0 | | | 0 | | | 0 | | 0 |
| *Pericrocotus roseus* | 0 | | 0 | 0 | | | 0 | | | 8 | | 0 | |
| *Pericrocotus solaris* | 0 | | 0 | 0 | | | 0 | | | 2 | | 4 | |
| *Pericrocotus brevirostris* | 0 | | 0 | 0 | | | 4 | | | 32 | | 0 | |
| *Pericrocotus flammeus* | 0 | | 15 | 14 | | | 0 | | | 0 | | 24 | |
| *Hemipus picatus* | 0 | | 0 | 0 | | | 0 | | | 0 | | 15 | |
| *Lanius collurioides* | 0 | | 0 | 4 | | | 0 | | | 0 | | 0 | |
| *Lanius schach* | 0 | | 6 | 7 | | | 1 | | | 3 | | 3 | |
| *Dicrurus macrocercus* | 0 | | 8 | 6 | | | 0 | | | 14 | | 18 | |
| *Dicrurus hottentottus* | 0 | | 0 | 0 | | | 0 | | | 0 | | 7 | |
| *Rhipidura hypoxantha* | 8 | | 8 | 4 | | | 9 | | | 0 | | 0 | |
| *Rhipidura albicollis* | 2 | | 4 | 0 | | | 2 | | | 1 | | 2 | |
| *Hypothymis azurea* | 0 | | 0 | 0 | | | 0 | | | 4 | | 2 | |
| *Urocissa erythrorhyncha* | 0 | | 6 | 0 | | | 0 | | | 3 | | 0 | |
| *Dendrocitta formosae* | 0 | | 0 | 0 | | | 0 | | | 0 | | 4 | |
| *Nucifraga caryocatactes* | 2 | | 0 | 0 | | | 0 | | | 0 | | 0 | |
| *Parus major* | 10 | | 11 | 8 | | | 0 | | | 11 | | 2 | |
| *Parus monticolus* | 6 | | 0 | 0 | | | 1 | | | 5 | | 1 | |
| *Parus spilonotus* | 8 | | 0 | 0 | | | 6 | | | 0 | | 2 | |
| *Sylviparus modestus* | 10 | | 0 | 0 | | | 16 | | | 0 | | 0 | |
| *Aegithalos concinnus* | 25 | | 14 | 17 | | | 10 | | | 16 | | 13 | |
| *Aegithalos iouschistos* | 0 | | 0 | 0 | | | 0 | | | 4 | | 0 | |
| *Prinia hodgsonii* | 0 | | 16 | 6 | | | 0 | | | 14 | | 3 | |
| *Spizixos canifrons* | 40 | | 0 | 0 | | | 55 | | | 0 | | 4 | |
| *Pycnonotus striatus* | 0 | | 0 | 0 | | | 0 | | | 0 | | 2 | |
| *Pycnonotus xanthorrhous* | 4 | | 25 | 10 | | | 0 | | | 12 | | 0 | |
| *Pycnonotus aurigaster* | 0 | | 10 | 16 | | | 0 | | | 18 | | 24 | |
| *Pycnonotus flavescens* | 0 | | 13 | 15 | | | 0 | | | 6 | | 6 | |
| *Hemixos flavala* | 0 | | 16 | 17 | | | 0 | | | 5 | | 11 | |
| *Hypsipetes mcclellandii* | 12 | | 6 | 0 | | | 6 | | | 0 | | 3 | |
| *Hypsipetes leucocephalus* | 9 | | 24 | 12 | | | 0 | | | 0 | | 0 | |
| *Orthotomus cuculatus* | 0 | | 2 | 10 | | | 1 | | | 3 | | 0 | |
| *Orthotomus sutorius* | 0 | | 4 | 0 | | | 0 | | | 8 | | 5 | |
| *Phylloscopus subaffinis* | 23 | | 4 | 0 | | | 3 | | | 0 | | 0 | |
| *Phylloscopus pulcher* | 0 | | 0 | 0 | | | 2 | | | 0 | | 0 | |
| *Phylloscopus maculipennis* | 0 | | 0 | 0 | | | 7 | | | 0 | | 0 | |
| *Phylloscopus inornatus* | 4 | | 15 | 0 | | | 0 | | | 0 | | 0 | |
| *Phylloscopus reguloides* | 0 | | 0 | 0 | | | 27 | | | 19 | | 32 | |
| *Seicercus burkii* | 0 | | 4 | 4 | | | 5 | | | 0 | | 0 | |
| *Seicercus castaniceps* | 0 | | 0 | 0 | | | 6 | | | 0 | | 0 | |
| *Abroscopus schisticeps* | 0 | 0 | | | 0 | 13 | | | 0 | | | 0 | |
| *Pomatorhinus gravivox* | 0 | 9 | | | 6 | 0 | | | 2 | | | 0 | |
| *Pomatorhinus ruficollis* | 6 | 12 | | | 18 | 1 | | | 9 | | | 2 | |
| *Pnoepyga pusilla* | 0 | 0 | | | 0 | 1 | | | 0 | | | 0 | |
| *Tesia castaneocoronata* | 0 | 0 | | | 0 | 1 | | | 0 | | | 0 | |
| *Tesia cyaniventer* | 0 | 0 | | | 0 | 1 | | | 0 | | | 0 | |
| *Stachyris ruficeps* | 0 | 0 | | | 0 | 3 | | | 0 | | | 0 | |
| *Babax lanceolatus* | 4 | 0 | | | 0 | 0 | | | 0 | | | 0 | |
| *Garrulax sannio* | 0 | 14 | | | 10 | 0 | | | 11 | | | 6 | |
| *Garrulax affinis* | 10 | 0 | | | 0 | 0 | | | 0 | | | 0 | |
| *Garrulax erythrocephalus* | 6 | 0 | | | 0 | 2 | | | 0 | | | 0 | |
| *Garrulax milnei* | 12 | 0 | | | 0 | 0 | | | 0 | | | 0 | |
| *Pteruthius flaviscapis* | 0 | 8 | | | 0 | 1 | | | 2 | | | 2 | |
| *Pteruthius xanthochlorus* | 0 | 0 | | | 0 | 11 | | | 0 | | | 0 | |
| *Pteruthius melanotis* | 6 | 0 | | | 0 | 0 | | | 0 | | | 0 | |
| *Actinodura souliei* | 4 | 0 | | | 0 | 2 | | | 0 | | | 0 | |
| *Minla cyanouroptera* | 0 | 14 | | | 0 | 10 | | | 0 | | | 2 | |
| *Minla strigula* | 37 | 0 | | | 0 | 19 | | | 0 | | | 0 | |
| *Minla ignotincta* | 19 | 0 | | | 0 | 7 | | | 0 | | | 0 | |
| *Alcippe chrysotis* | 28 | 0 | | | 0 | 5 | | | 0 | | | 0 | |
| *Alcippe castaneceps* | 0 | 0 | | | 0 | 18 | | | 0 | | | 0 | |
| *Alcippe cinereiceps* | 0 | 0 | | | 0 | 4 | | | 0 | | | 0 | |
| *Alcippe dubia* | 6 | 0 | | | 0 | 1 | | | 0 | | | 0 | |
| *Alcippe poioicephala* | 4 | 0 | | | 0 | 0 | | | 0 | | | 0 | |
| *Alcippe morrisonia* | 28 | 0 | | | 0 | 26 | | | 0 | | | 17 | |
| *Heterophasia desgodinsi* | 10 | 0 | | | 0 | 12 | | | 0 | | | 5 | |
| *Yuhina flavicollis* | 39 | 0 | | | 0 | 11 | | | 0 | | | 6 | |
| *Yuhina gularis* | 20 | 0 | | | 0 | 21 | | | 0 | | | 0 | |
| *Yuhina diademata* | 23 | 0 | | | 0 | 4 | | | 0 | | | 0 | |
| *Yuhina occipitalis* | 24 | 0 | | | 0 | 15 | | | 0 | | | 0 | |
| *Erpornis zantholeuca* | 0 | 0 | | | 0 | 0 | | | 0 | | | 6 | |
| *Paradoxornis nipalensis* | 0 | 0 | | | 0 | 6 | | | 0 | | | 0 | |
| *Sitta nagaensis* | 0 | 0 | | | 0 | 0 | | | 1 | | | 2 | |
| *Zosterops japonicus* | 0 | 45 | | | 30 | 0 | | | 45 | | | 44 | |
| *Sitta himalayensis* | 8 | 0 | | | 0 | 5 | | | 0 | | | 0 | |
| *Sitta yunnanensis* | 0 | 0 | | | 0 | 0 | | | 2 | | | 0 | |
| *Sitta frontalis* | 0 | 0 | | | 0 | 0 | | | 2 | | | 5 | |
| *Sitta formosa* | 0 | 0 | | | 0 | 0 | | | 1 | | | 0 | |
| *Myophonus caeruleus* | 2 | 0 | | | 0 | 0 | | | 0 | | | 0 | |
| *Turdus rubrocanus* | 14 | 0 | | | 0 | 0 | | | 0 | | | 0 | |
| *Turdus mupinensis* | 4 | 0 | | | 0 | 0 | | | 0 | | | 0 | |
| *Tarsiger cyanurus* | 4 | 4 | | | 0 | 0 | | | 0 | | | 0 | |
| *Tarsiger chrysaeus* | 0 | 0 | | | 0 | 1 | | | 0 | | | 0 | |
| *Cinclidium leucurum* | 0 | 0 | | | 0 | 2 | | | 0 | | | 0 | |
| *Copsychus saularis* | 0 | 8 | | | 5 | 0 | | | 4 | | | 3 | |
| *Phoenicurus auroreus* | 0 | 2 | | | 4 | 0 | | | 0 | | | 0 | |
| *Phoenicurus frontalis* | 4 | 0 | | | 0 | 0 | | | 0 | | | 0 | |
| *Rhyacornis fuliginosa* | 7 | 3 | | | 5 | 5 | | | 0 | | | 0 | |
| *Chaimarrornis leucocephalus* | 0 | 0 | | | 2 | 0 | | | 0 | | | 0 | |
| *Enicurus leschenaulti* | 0 | 0 | | | 0 | 1 | | | 0 | | | 0 | |
| *Enicurus schistaceus* | 0 | 0 | | | 4 | 0 | | | 1 | | | 1 | |
| *Enicurus maculatus* | 2 | 0 | | | 0 | 1 | | | 0 | | | 0 | |
| *Monticola solitarius* | 0 | 0 | | | 0 | 0 | | | 0 | | | 1 | |
| *Saxicola torquatus* | 8 | 0 | | | 0 | 3 | | | 0 | | | 0 | |
| *Saxicola caprata* | 0 | 0 | | | 6 | 0 | | | 0 | | | 0 | |
| *Muscicapa sibirica* | 0 | 0 | | | 0 | 1 | | | 1 | | | 0 | |
| *Saxicola ferreus* | 0 | 12 | | | 4 | 0 | | | 7 | | | 0 | |
| *Monticola rufiventris* | 2 | 0 | | | 0 | 0 | | | 0 | | | 0 | |
| *Ficedula hyperythra* | 0 | 0 | | | 0 | 6 | | | 0 | | | 0 | |
| *Ficedula tricolor* | 0 | 0 | | | 0 | 1 | | | 0 | | | 0 | |
| *Cyornis rubeculoides* | 0 | 0 | | | 0 | 0 | | | 8 | | | 0 | |
| *Ficedula westermanni* | 0 | 0 | | | 2 | 0 | | | 0 | | | 0 | |
| *Niltava sundara* | 0 | 0 | | | 0 | 4 | | | 1 | | | 0 | |
| *Eumyias thalassinus* | 0 | 2 | | | 0 | 0 | | | 0 | | | 0 | |
| *Culicicapa ceylonensis* | 0 | 0 | | | 0 | 4 | | | 9 | | | 12 | |
| *Cyornis banyumas* | 0 | 2 | | | 0 | 0 | | | 0 | | | 0 | |
| *Chloropsis cochinchinensis* | 0 | 0 | | | 0 | 0 | | | 3 | | | 0 | |
| *Aethopyga gouldiae* | 0 | 0 | | | 0 | 1 | | | 0 | | | 0 | |
| *Chloropsis hardwickii* | 0 | 5 | | | 3 | 0 | | | 3 | | | 0 | |
| *Dicaeum melanoxanthum* | 0 | 4 | | | 0 | 0 | | | 0 | | | 0 | |
| *Aethopyga nipalensis* | 0 | 0 | | | 0 | 1 | | | 0 | | | 0 | |
| *Dicaeum ignipectus* | 0 | 6 | | | 4 | 5 | | | 0 | | | 5 | |
| *Aethopyga saturata* | 0 | 0 | | | 2 | 0 | | | 0 | | | 0 | |
| *Aethopyga siparaja* | 0 | 6 | | | 0 | 0 | | | 2 | | | 0 | |
| *Lonchura striata* | 0 | 0 | | | 19 | 0 | | | 16 | | | 7 | |
| *Arachnothera magna* | 0 | 2 | | | 0 | 0 | | | 0 | | | 1 | |
| *Passer rutilans* | 0 | 12 | | | 0 | 0 | | | 25 | | | 0 | |
| *Leiothrix argentauris* | 0 | 0 | | | 0 | 0 | | | 0 | | | 4 | |
| *Lonchura punctulata* | 0 | 0 | | | 16 | 0 | | | 0 | | | 3 | |
| *Motacilla alba* | 5 | 9 | | | 5 | 4 | | | 5 | | | 8 | |
| *Motacilla cinerea* | 0 | 0 | | | 2 | 0 | | | 0 | | | 0 | |
| *Anthus hodgsoni* | 16 | 7 | | | 8 | 0 | | | 0 | | | 0 | |
| *Carduelis ambigua* | 0 | 11 | | | 0 | 0 | | | 13 | | | 0 | |
| *Pinicola subhimachala* | 7 | 0 | | | 0 | 0 | | | 0 | | | 0 | |
| *Emberiza fucata* | 5 | 10 | | | 0 | 0 | | | 0 | | | 0 | |

The plot names were [abbreviated](javascript:void(0);) (XJB: Xujiaba, PZ: Pizhang and DDY: Daduanyao)[, and](javascript:void(0);) with a -W and -B appended represented wintering (-W) season and breeding (-B) season.

The 14 additional non-passerine species were: *Lophura nycthemera, Streptopelia orientalis, Phaenicophaeus tristis, Accipiter trivirgatus, Buteo buteo, Psilopogon asiaticus, Dryobates pernyii* and *Dendrocopos darjellensis* in wintering season and *Streptopelia orientalis, Phaenicophaeus tristis, Accipiter virgatus, Psilopogon virens, P. asiaticus, Picumnus innominatus, Micropternus brachyurus, Picoides canicapillus, Dendrocopos darjellensis* and *Psittacula finschii* in breeding season.

**TABLE S3** Species × trait data matrix for the 125 passerine birds of three elevational gradient plots on Ailao Mountains.

|  | **Bodymass^a^** | **Generation length^b^** | **Migratory status** | **Seeds** | **Nectar** | **Freshy fruits** | **Invertebrates** | **Veterbrate** | **Glean** | **Pobe** | **Sally** | **Leap** | **Water** | **Ground** | **Understory** | **Midstory** | **Canopy** |
| --- | --- | --- | --- | --- | --- | --- | --- | --- | --- | --- | --- | --- | --- | --- | --- | --- | --- |
| ***Coracina melaschistos*** | 43 | 4.6 | 1 | 0 | 0 | 1 | 1 | 0 | 1 | 0 | 1 | 0 | 0 | 0 | 0 | 0 | 1 |
| ***Pericrocotus roseus*** | 19.2 | 4.6 | 1 | 0 | 0 | 0 | 1 | 0 | 0 | 1 | 1 | 0 | 0 | 0 | 0 | 0 | 1 |
| ***Pericrocotus solaris*** | 14.5 | 4.6 | 1 | 0 | 0 | 0 | 1 | 0 | 0 | 1 | 1 | 0 | 0 | 0 | 0 | 0 | 1 |
| ***Pericrocotus brevirostris*** | 16.5 | 4.6 | 1 | 0 | 0 | 0 | 1 | 0 | 0 | 1 | 1 | 0 | 0 | 0 | 0 | 0 | 1 |
| ***Pericrocotus flammeus*** | 23.3 | 4.6 | 0 | 0 | 0 | 0 | 1 | 0 | 0 | 1 | 1 | 0 | 0 | 0 | 0 | 0 | 1 |
| ***Hemipus picatus*** | 10.5 | 4.3 | 0 | 0 | 0 | 0 | 1 | 0 | 0 | 1 | 1 | 0 | 0 | 0 | 0 | 0 | 1 |
| ***Lanius collurioides*** | 28.5 | 3.9 | 1 | 0 | 0 | 0 | 1 | 1 | 0 | 0 | 1 | 0 | 0 | 1 | 1 | 0 | 0 |
| ***Lanius schach*** | 51.6 | 3.6 | 1 | 0 | 0 | 0 | 1 | 1 | 0 | 0 | 1 | 0 | 0 | 1 | 1 | 0 | 0 |
| ***Dicrurus macrocercus*** | 48.3 | 7.9 | 1 | 0 | 0 | 0 | 1 | 0 | 0 | 0 | 1 | 0 | 0 | 0 | 0 | 0 | 1 |
| ***Dicrurus hottentottus*** | 79.2 | 7.9 | 1 | 0 | 0 | 0 | 1 | 0 | 0 | 0 | 1 | 0 | 0 | 0 | 0 | 0 | 1 |
| ***Rhipidura hypoxantha*** | 5.5 | 4.9 | 1 | 0 | 0 | 0 | 1 | 0 | 0 | 0 | 1 | 1 | 0 | 0 | 1 | 1 | 0 |
| ***Rhipidura albicollis*** | 12.9 | 4.9 | 1 | 0 | 0 | 0 | 1 | 0 | 0 | 0 | 1 | 1 | 0 | 0 | 1 | 1 | 0 |
| ***Hypothymis azurea*** | 11.1 | 4.2 | 1 | 0 | 0 | 0 | 1 | 0 | 0 | 0 | 1 | 1 | 0 | 0 | 0 | 1 | 0 |
| ***Urocissa erythrorhyncha*** | 160.4 | 6.7 | 1 | 1 | 0 | 1 | 1 | 1 | 1 | 1 | 1 | 0 | 0 | 1 | 1 | 1 | 1 |
| ***Dendrocitta formosae*** | 97.5 | 6.7 | 0 | 1 | 0 | 1 | 0 | 0 | 1 | 0 | 0 | 0 | 0 | 0 | 0 | 1 | 1 |
| ***Nucifraga caryocatactes*** | 194.5 | 7.5 | 0 | 1 | 0 | 1 | 1 | 0 | 1 | 0 | 0 | 0 | 0 | 0 | 0 | 1 | 1 |
| ***Parus major*** | 18.4 | 4.3 | 0 | 0 | 0 | 0 | 1 | 0 | 1 | 1 | 0 | 0 | 0 | 0 | 1 | 1 | 0 |
| ***Parus monticolus*** | 14.1 | 4.2 | 1 | 0 | 0 | 0 | 1 | 0 | 1 | 1 | 0 | 0 | 0 | 0 | 1 | 1 | 0 |
| ***Parus spilonotus*** | 18.8 | 4.2 | 1 | 0 | 0 | 0 | 1 | 0 | 1 | 1 | 0 | 0 | 0 | 0 | 0 | 1 | 1 |
| ***Sylviparus modestus*** | 7.3 | 5.2 | 1 | 0 | 0 | 0 | 1 | 0 | 1 | 1 | 0 | 0 | 0 | 0 | 1 | 1 | 0 |
| ***Aegithalos concinnus*** | 6.1 | 4.2 | 0 | 0 | 0 | 0 | 1 | 0 | 1 | 1 | 0 | 1 | 0 | 0 | 1 | 1 | 0 |
| ***Aegithalos iouschistos*** | 6.9 | 4.2 | 1 | 1 | 0 | 0 | 1 | 0 | 1 | 0 | 0 | 1 | 0 | 0 | 1 | 1 | 0 |
| ***Prinia hodgsonii*** | 6.4 | 3.8 | 1 | 0 | 0 | 0 | 1 | 0 | 1 | 0 | 0 | 1 | 0 | 0 | 1 | 0 | 0 |
| ***Spizixos canifrons*** | 44 | 3.7 | 0 | 1 | 0 | 1 | 1 | 0 | 1 | 0 | 1 | 0 | 0 | 0 | 1 | 1 | 0 |
| ***Pycnonotus striatus*** | 51.8 | 3.7 | 1 | 1 | 0 | 1 | 0 | 0 | 1 | 0 | 0 | 0 | 0 | 0 | 0 | 1 | 1 |
| ***Pycnonotus xanthorrhous*** | 26.9 | 3.7 | 0 | 1 | 0 | 1 | 0 | 0 | 1 | 0 | 0 | 0 | 0 | 0 | 0 | 1 | 1 |
| ***Pycnonotus aurigaster*** | 44.4 | 3.7 | 0 | 1 | 0 | 1 | 1 | 0 | 1 | 0 | 0 | 0 | 0 | 0 | 0 | 1 | 1 |
| ***Pycnonotus flavescens*** | 28.9 | 3.7 | 1 | 1 | 0 | 1 | 0 | 0 | 1 | 0 | 0 | 0 | 0 | 0 | 0 | 1 | 1 |
| ***Hemixos flavala*** | 32.5 | 3.5 | 1 | 1 | 0 | 1 | 0 | 0 | 1 | 0 | 0 | 0 | 0 | 0 | 1 | 0 | 1 |
| ***Hypsipetes mcclellandii*** | 32.5 | 3.5 | 1 | 1 | 0 | 1 | 0 | 0 | 1 | 0 | 0 | 0 | 0 | 0 | 1 | 0 | 1 |
| ***Hypsipetes leucocephalus*** | 52.5 | 3.5 | 1 | 0 | 1 | 1 | 1 | 0 | 1 | 0 | 1 | 0 | 0 | 0 | 0 | 0 | 1 |
| ***Orthotomus cuculatus*** | 5.9 | 3.6 | 1 | 0 | 0 | 0 | 1 | 0 | 0 | 1 | 0 | 1 | 0 | 0 | 1 | 1 | 0 |
| ***Orthotomus sutorius*** | 7.5 | 3.6 | 0 | 0 | 0 | 0 | 1 | 0 | 0 | 1 | 0 | 1 | 0 | 0 | 1 | 1 | 0 |
| ***Phylloscopus subaffinis*** | 6.2 | 3.6 | 1 | 0 | 0 | 0 | 1 | 0 | 0 | 1 | 0 | 1 | 0 | 0 | 1 | 0 | 0 |
| ***Phylloscopus pulcher*** | 6.8 | 3.6 | 1 | 0 | 0 | 0 | 1 | 0 | 0 | 0 | 0 | 1 | 0 | 0 | 1 | 0 | 1 |
| ***Phylloscopus maculipennis*** | 5.1 | 3.6 | 1 | 0 | 0 | 0 | 1 | 0 | 0 | 1 | 0 | 1 | 0 | 0 | 0 | 1 | 1 |
| ***Phylloscopus inornatus*** | 6.4 | 3.6 | 1 | 1 | 0 | 0 | 1 | 0 | 1 | 0 | 0 | 1 | 0 | 0 | 1 | 1 | 1 |
| ***Phylloscopus reguloides*** | 8 | 3.6 | 1 | 0 | 0 | 0 | 1 | 0 | 0 | 1 | 0 | 1 | 0 | 0 | 0 | 0 | 1 |
| ***Seicercus burkii*** | 7.3 | 3.6 | 1 | 0 | 0 | 0 | 1 | 0 | 0 | 1 | 1 | 0 | 0 | 0 | 1 | 1 | 0 |
| ***Seicercus castaniceps*** | 5.3 | 3.6 | 1 | 0 | 0 | 0 | 1 | 0 | 0 | 1 | 0 | 0 | 0 | 0 | 1 | 1 | 0 |
| ***Abroscopus schisticeps*** | 4.7 | 3.6 | 0 | 0 | 0 | 0 | 1 | 0 | 0 | 1 | 0 | 0 | 0 | 0 | 1 | 1 | 0 |
| ***Pomatorhinus gravivox*** | 62.5 | 6.5 | 0 | 0 | 0 | 0 | 1 | 0 | 1 | 1 | 0 | 0 | 0 | 1 | 1 | 0 | 0 |
| ***Pomatorhinus ruficollis*** | 32.0 | 5.5 | 0 | 0 | 0 | 0 | 1 | 0 | 1 | 1 | 0 | 0 | 0 | 1 | 1 | 0 | 0 |
| ***Pnoepyga pusilla*** | 12 | 4.4 | 1 | 0 | 0 | 0 | 1 | 0 | 1 | 1 | 0 | 0 | 0 | 1 | 1 | 0 | 0 |
| ***Tesia castaneocoronata*** | 8 | 3.6 | 1 | 0 | 0 | 0 | 1 | 0 | 0 | 1 | 0 | 1 | 0 | 1 | 1 | 0 | 0 |
| ***Tesia cyaniventer*** | 9.7 | 3.6 | 1 | 0 | 0 | 0 | 1 | 0 | 0 | 1 | 0 | 1 | 0 | 1 | 1 | 0 | 0 |
| ***Stachyris ruficeps*** | 10.3 | 3.7 | 0 | 0 | 0 | 0 | 1 | 0 | 0 | 1 | 0 | 0 | 0 | 1 | 1 | 0 | 0 |
| ***Babax lanceolatus*** | 75.5 | 5.5 | 0 | 1 | 0 | 0 | 1 | 0 | 0 | 1 | 0 | 0 | 0 | 1 | 1 | 0 | 0 |
| ***Garrulax sannio*** | 67.8 | 4.7 | 0 | 1 | 0 | 1 | 1 | 0 | 1 | 1 | 0 | 0 | 0 | 1 | 1 | 0 | 0 |
| ***Garrulax affinis*** | 73 | 4.7 | 1 | 1 | 0 | 1 | 1 | 0 | 1 | 1 | 0 | 0 | 0 | 1 | 1 | 0 | 0 |
| ***Garrulax erythrocephalus*** | 71.7 | 4.7 | 0 | 1 | 0 | 1 | 1 | 0 | 0 | 1 | 0 | 0 | 0 | 1 | 1 | 0 | 0 |
| ***Garrulax milnei*** | 79.8 | 4.7 | 0 | 0 | 0 | 1 | 1 | 0 | 0 | 1 | 0 | 0 | 0 | 1 | 1 | 0 | 0 |
| ***Pteruthius flaviscapis*** | 39 | 5.1 | 1 | 0 | 0 | 0 | 1 | 0 | 1 | 0 | 0 | 0 | 0 | 0 | 0 | 1 | 0 |
| ***Pteruthius xanthochlorus*** | 14.3 | 5.1 | 1 | 0 | 0 | 0 | 1 | 0 | 1 | 0 | 0 | 0 | 0 | 0 | 0 | 1 | 1 |
| ***Pteruthius melanotis*** | 13.3 | 5.1 | 0 | 0 | 0 | 0 | 1 | 0 | 1 | 1 | 0 | 0 | 0 | 0 | 0 | 1 | 1 |
| ***Actinodura souliei*** | 55.5 | 5.5 | 0 | 0 | 0 | 0 | 1 | 0 | 1 | 1 | 0 | 0 | 0 | 0 | 1 | 1 | 0 |
| ***Minla cyanouroptera*** | 17 | 4.4 | 1 | 0 | 0 | 0 | 1 | 0 | 1 | 1 | 0 | 0 | 0 | 0 | 0 | 1 | 0 |
| ***Minla strigula*** | 19.2 | 4.4 | 0 | 0 | 0 | 0 | 1 | 0 | 1 | 1 | 0 | 0 | 0 | 0 | 0 | 1 | 1 |
| ***Minla ignotincta*** | 14.3 | 4.4 | 0 | 0 | 0 | 0 | 1 | 0 | 1 | 1 | 0 | 0 | 0 | 0 | 0 | 1 | 1 |
| ***Alcippe chrysotis*** | 8.5 | 3.8 | 0 | 0 | 0 | 0 | 1 | 0 | 1 | 1 | 0 | 0 | 0 | 0 | 1 | 0 | 0 |
| ***Alcippe castaneceps*** | 12.5 | 3.8 | 0 | 0 | 0 | 0 | 1 | 0 | 1 | 1 | 0 | 0 | 0 | 0 | 1 | 1 | 0 |
| ***Alcippe cinereiceps*** | 11.3 | 3.8 | 0 | 0 | 0 | 0 | 1 | 0 | 1 | 1 | 0 | 0 | 0 | 0 | 1 | 0 | 0 |
| ***Alcippe dubia*** | 18.5 | 3.8 | 0 | 0 | 0 | 0 | 1 | 0 | 1 | 1 | 0 | 0 | 0 | 0 | 1 | 0 | 0 |
| ***Alcippe poioicephala*** | 20.7 | 3.8 | 0 | 1 | 0 | 1 | 1 | 0 | 1 | 0 | 0 | 0 | 0 | 0 | 1 | 0 | 0 |
| ***Alcippe morrisonia*** | 14.8 | 3.8 | 0 | 0 | 0 | 0 | 1 | 0 | 1 | 1 | 0 | 0 | 0 | 0 | 1 | 1 | 0 |
| ***Heterophasia desgodinsi*** | 40.8 | 4.9 | 0 | 0 | 0 | 0 | 1 | 0 | 1 | 0 | 0 | 0 | 0 | 0 | 0 | 1 | 1 |
| ***Yuhina flavicollis*** | 17.5 | 5.7 | 1 | 0 | 0 | 1 | 1 | 0 | 1 | 0 | 0 | 0 | 0 | 0 | 1 | 1 | 0 |
| ***Yuhina gularis*** | 21 | 5.7 | 1 | 1 | 1 | 1 | 0 | 0 | 1 | 0 | 0 | 0 | 0 | 0 | 1 | 1 | 0 |
| ***Yuhina diademata*** | 22 | 5.7 | 0 | 1 | 0 | 1 | 1 | 0 | 1 | 0 | 0 | 0 | 0 | 0 | 0 | 1 | 1 |
| ***Yuhina occipitalis*** | 13 | 5.7 | 1 | 0 | 0 | 0 | 1 | 0 | 1 | 0 | 0 | 0 | 0 | 0 | 1 | 1 | 1 |
| ***Erpornis zantholeuca*** | 11.8 | 4.4 | 0 | 0 | 0 | 0 | 1 | 0 | 1 | 0 | 0 | 0 | 0 | 0 | 0 | 1 | 1 |
| ***Paradoxornis nipalensis*** | 6 | 4.6 | 0 | 1 | 0 | 0 | 1 | 0 | 1 | 0 | 0 | 1 | 0 | 0 | 1 | 0 | 0 |
| ***Sitta nagaensis*** | 14.7 | 4 | 0 | 0 | 0 | 0 | 1 | 0 | 0 | 1 | 0 | 0 | 0 | 0 | 0 | 0 | 1 |
| ***Zosterops japonicus*** | 11.1 | 3.5 | 1 | 1 | 0 | 1 | 1 | 0 | 1 | 0 | 0 | 0 | 0 | 0 | 0 | 1 | 1 |
| ***Sitta himalayensis*** | 14.5 | 4 | 0 | 0 | 0 | 0 | 1 | 0 | 0 | 1 | 0 | 0 | 0 | 0 | 0 | 0 | 1 |
| ***Sitta yunnanensis*** | 11 | 4 | 1 | 0 | 0 | 0 | 1 | 0 | 0 | 1 | 0 | 0 | 0 | 0 | 0 | 0 | 1 |
| ***Sitta frontalis*** | 16.5 | 4 | 0 | 0 | 0 | 0 | 1 | 0 | 0 | 1 | 0 | 0 | 0 | 0 | 0 | 0 | 1 |
| ***Sitta formosa*** | 34.5 | 4 | 1 | 0 | 0 | 0 | 1 | 0 | 0 | 1 | 0 | 0 | 0 | 0 | 0 | 0 | 1 |
| ***Myophonus caeruleus*** | 157.5 | 4.3 | 1 | 1 | 0 | 1 | 1 | 0 | 1 | 0 | 0 | 0 | 1 | 1 | 0 | 0 | 0 |
| ***Turdus rubrocanus*** | 92.2 | 6.1 | 1 | 0 | 0 | 1 | 1 | 0 | 0 | 1 | 0 | 0 | 0 | 1 | 1 | 0 | 0 |
| ***Turdus mupinensis*** | 60.8 | 6.1 | 0 | 0 | 0 | 1 | 1 | 0 | 1 | 1 | 0 | 0 | 0 | 1 | 1 | 0 | 0 |
| ***Tarsiger cyanurus*** | 14 | 3.8 | 1 | 1 | 0 | 1 | 1 | 0 | 1 | 0 | 1 | 0 | 0 | 1 | 1 | 0 | 0 |
| ***Tarsiger chrysaeus*** | 13.8 | 3.8 | 1 | 0 | 0 | 0 | 1 | 0 | 0 | 1 | 0 | 0 | 0 | 1 | 0 | 0 | 0 |
| ***Cinclidium leucurum*** | 24.8 | 3.8 | 0 | 0 | 0 | 0 | 1 | 0 | 0 | 1 | 1 | 0 | 0 | 1 | 1 | 0 | 0 |
| ***Copsychus saularis*** | 36 | 3.6 | 0 | 0 | 0 | 0 | 1 | 0 | 1 | 0 | 0 | 0 | 0 | 1 | 1 | 0 | 0 |
| ***Phoenicurus auroreus*** | 16.2 | 4.1 | 1 | 0 | 0 | 0 | 1 | 0 | 0 | 0 | 1 | 0 | 0 | 0 | 1 | 1 | 0 |
| ***Phoenicurus frontalis*** | 16 | 4.1 | 1 | 0 | 0 | 0 | 1 | 0 | 0 | 0 | 1 | 0 | 0 | 1 | 1 | 0 | 0 |
| ***Rhyacornis fuliginosa*** | 21 | 3.8 | 0 | 0 | 0 | 0 | 1 | 0 | 0 | 1 | 1 | 0 | 1 | 1 | 0 | 0 | 0 |
| ***Chaimarrornis leucocephalus*** | 30.4 | 3.8 | 1 | 0 | 0 | 0 | 1 | 0 | 0 | 1 | 1 | 0 | 1 | 1 | 0 | 0 | 0 |
| ***Enicurus leschenaulti*** | 33.8 | 3.8 | 0 | 0 | 0 | 0 | 1 | 0 | 0 | 1 | 1 | 0 | 1 | 1 | 0 | 0 | 0 |
| ***Enicurus schistaceus*** | 31 | 3.8 | 0 | 0 | 0 | 0 | 1 | 0 | 0 | 1 | 1 | 0 | 1 | 1 | 0 | 0 | 0 |
| ***Enicurus maculatus*** | 41 | 3.8 | 0 | 0 | 0 | 0 | 1 | 0 | 0 | 1 | 1 | 0 | 1 | 1 | 0 | 0 | 0 |
| ***Monticola solitarius*** | 50.5 | 3.8 | 1 | 0 | 0 | 0 | 1 | 0 | 0 | 0 | 1 | 0 | 0 | 1 | 0 | 0 | 0 |
| ***Saxicola torquatus*** | 15.2 | 4.1 | 1 | 0 | 0 | 0 | 1 | 0 | 0 | 0 | 0 | 1 | 0 | 0 | 1 | 0 | 0 |
| ***Saxicola caprata*** | 15.2 | 4.1 | 1 | 0 | 0 | 0 | 1 | 0 | 0 | 0 | 0 | 1 | 0 | 0 | 1 | 0 | 0 |
| ***Muscicapa sibirica*** | 13.2 | 3.8 | 1 | 0 | 0 | 0 | 1 | 0 | 0 | 1 | 0 | 0 | 0 | 0 | 0 | 0 | 1 |
| ***Saxicola ferreus*** | 14.7 | 4.1 | 0 | 0 | 0 | 0 | 1 | 0 | 0 | 0 | 0 | 1 | 0 | 0 | 1 | 0 | 0 |
| ***Monticola rufiventris*** | 53.2 | 3.8 | 1 | 0 | 0 | 0 | 1 | 0 | 0 | 1 | 0 | 0 | 0 | 1 | 0 | 0 | 0 |
| ***Ficedula hyperythra*** | 8.2 | 3.9 | 0 | 0 | 0 | 0 | 1 | 0 | 0 | 0 | 1 | 0 | 0 | 0 | 1 | 0 | 0 |
| ***Ficedula tricolor*** | 8.4 | 3.9 | 1 | 0 | 0 | 0 | 1 | 0 | 0 | 0 | 0 | 1 | 0 | 0 | 1 | 0 | 0 |
| ***Cyornis rubeculoides*** | 14.2 | 3.8 | 1 | 0 | 0 | 0 | 1 | 0 | 0 | 0 | 0 | 1 | 0 | 0 | 1 | 0 | 0 |
| ***Ficedula westermanni*** | 7.8 | 3.9 | 0 | 0 | 0 | 0 | 1 | 0 | 0 | 0 | 0 | 1 | 0 | 0 | 1 | 0 | 0 |
| ***Niltava sundara*** | 21.2 | 3.8 | 0 | 0 | 0 | 0 | 1 | 0 | 0 | 0 | 0 | 1 | 0 | 0 | 1 | 0 | 0 |
| ***Eumyias thalassinus*** | 18.1 | 3.8 | 1 | 0 | 0 | 0 | 1 | 0 | 0 | 0 | 1 | 0 | 0 | 0 | 0 | 0 | 1 |
| ***Culicicapa ceylonensis*** | 8.8 | 3.8 | 0 | 0 | 0 | 0 | 1 | 0 | 0 | 0 | 0 | 1 | 0 | 0 | 0 | 1 | 0 |
| ***Cyornis banyumas*** | 14.5 | 3.8 | 0 | 0 | 0 | 0 | 1 | 0 | 0 | 0 | 0 | 1 | 0 | 0 | 1 | 0 | 0 |
| ***Chloropsis cochinchinensis*** | 24.5 | 4.2 | 0 | 0 | 0 | 0 | 1 | 0 | 0 | 1 | 0 | 0 | 0 | 0 | 0 | 0 | 1 |
| ***Aethopyga gouldiae*** | 7.3 | 4.2 | 0 | 0 | 1 | 0 | 0 | 0 | 0 | 1 | 0 | 0 | 0 | 0 | 0 | 1 | 1 |
| ***Chloropsis hardwickii*** | 32.8 | 4.2 | 0 | 0 | 0 | 0 | 1 | 0 | 0 | 1 | 0 | 0 | 0 | 0 | 0 | 0 | 1 |
| ***Dicaeum melanoxanthum*** | 14.3 | 5.7 | 1 | 1 | 0 | 1 | 1 | 0 | 1 | 0 | 0 | 0 | 0 | 0 | 0 | 0 | 1 |
| ***Aethopyga nipalensis*** | 6.5 | 4.2 | 0 | 0 | 1 | 0 | 0 | 0 | 0 | 1 | 0 | 0 | 0 | 0 | 0 | 1 | 1 |
| ***Dicaeum ignipectus*** | 5.9 | 5.7 | 0 | 0 | 0 | 1 | 1 | 0 | 1 | 0 | 0 | 0 | 0 | 0 | 0 | 0 | 1 |
| ***Aethopyga saturata*** | 6.8 | 4.2 | 0 | 1 | 1 | 0 | 1 | 0 | 1 | 0 | 0 | 0 | 0 | 0 | 1 | 1 | 0 |
| ***Aethopyga siparaja*** | 6.5 | 4.2 | 0 | 0 | 1 | 0 | 1 | 0 | 0 | 1 | 0 | 0 | 0 | 0 | 0 | 1 | 1 |
| ***Lonchura striata*** | 12.3 | 3.8 | 0 | 1 | 0 | 1 | 0 | 0 | 1 | 0 | 0 | 0 | 0 | 0 | 1 | 1 | 0 |
| ***Arachnothera magna*** | 30.7 | 4.2 | 0 | 0 | 0 | 0 | 1 | 0 | 0 | 1 | 0 | 0 | 0 | 0 | 0 | 1 | 1 |
| ***Passer rutilans*** | 18.3 | 5.8 | 1 | 1 | 0 | 1 | 1 | 0 | 1 | 0 | 0 | 0 | 0 | 0 | 1 | 1 | 0 |
| ***Leiothrix argentauris*** | 28.4 | 5.6 | 0 | 0 | 0 | 0 | 1 | 0 | 1 | 1 | 0 | 0 | 0 | 1 | 1 | 0 | 0 |
| ***Lonchura punctulata*** | 13.6 | 3.8 | 0 | 1 | 0 | 1 | 0 | 0 | 1 | 0 | 0 | 0 | 0 | 0 | 1 | 1 | 0 |
| ***Motacilla alba*** | 23.6 | 4.2 | 1 | 0 | 0 | 0 | 1 | 0 | 0 | 0 | 1 | 0 | 1 | 1 | 0 | 0 | 0 |
| ***Motacilla cinerea*** | 17.8 | 4.7 | 1 | 0 | 0 | 0 | 1 | 0 | 0 | 0 | 1 | 0 | 1 | 1 | 0 | 0 | 0 |
| ***Anthus hodgsoni*** | 21.3 | 3.7 | 1 | 0 | 0 | 0 | 1 | 0 | 0 | 0 | 1 | 0 | 0 | 1 | 0 | 0 | 0 |
| ***Carduelis ambigua*** | 17.5 | 4.2 | 1 | 1 | 0 | 1 | 0 | 0 | 1 | 0 | 0 | 0 | 0 | 0 | 0 | 1 | 1 |
| ***Pinicola subhimachala*** | 46.2 | 4.2 | 1 | 1 | 0 | 0 | 0 | 0 | 1 | 0 | 0 | 0 | 0 | 0 | 0 | 1 | 1 |
| ***Emberiza fucata*** | 19.8 | 3.6 | 1 | 1 | 0 | 1 | 1 | 0 | 1 | 0 | 0 | 0 | 0 | 0 | 1 | 0 | 0 |

a: For the body mass information absent from CRC handbook of avian body masses (Dunning, 2007), twenty-four were extracted from A handbook of the birds of China (Zhao, 2001), one (*Pomatorhinus gravivox*) was collected from Handbook of the birds of the world Alive (del Hoyo, Elliott, Sargatal, Christie, & de Juana, 2015).

The 24 species were listed below:

*Hemipus picatus, Lanius collurioides, Dendrocitta formosae, Aegithalos iouschistos, Pycnonotus striatus, Phylloscopus reguloides, Tesia castaneocoronata, T. cyaniventer, Babax lanceolatus, Garrulax milnei, Actinodura souliei, A. chrysotis, A. cinereiceps, A. dubia, Heterophasia desgodinsi, Yuhina diademata, Cinclidium leucurum, Culicicapa ceylonensis, Dicaeum melanoxanthum, Aethopyga gouldiae, A. nipalensis, A. saturata, A. siparaja, Carduelis ambigua.*

b: The generation length information were all retrieved from BirdLife International’s World Bird Database (available online at http://www.birdlife.org/datazone/home), but there were 30 species which the generation length lacked in the database, even relative species in the same genus, so the data were instead by the mean value of all species belong to the nearest genus (TABLE S3-b).

TABLE S3-b. Generation length source information of the 30 species which lacked in BirdLife International’s World Bird Database.

| **Species** | **Reference genus** | | **Generation length** | |
| --- | --- | --- | --- | --- |
| *Pericrocotus roseus; P. solaris; P. brevirostris; P. flammeus* | Campephaga; n=6^*^ | | 4.6 | |
| *Hemipus picatus* | Daphoenositta; n=2 | | 4.3 | |
| *Dicrurus macrocercus; D. hottentottus* | Oreoica; n=1 | | 7.9 | |
| *Spizixos canifrons; Pycnonotus striatus; P. xanthorrhous; P. aurigaster; P. flavescens* | Andropadus; n=12 | 3.7 | |  |
| *Hypsipetes mcclellandii; H. leucocephalus* | Hemixos; n=2 | | 3.5 | |
| *Orthotomus cuculatus; O. sutorius* | Artisornis; n=2 | | 3.6 | |
| *Muscicapa sibirica* | Rhinomyias; n=11 | | 3.8 | |
| *Saxicola torquatus; S. caprata; S. ferreus* | Oenanthe; n=23 | | 4.1 | |
| *Seicercus burkii; S. castaniceps* | Phylloscopus; n=2 | | 3.6 | |
| *Cyornis rubeculoides; C. banyumas*  *Niltava sundara; Culicicapa ceylonensis* | Eumyias; n=5 | | 3.8 | |
| *Dicaeum melanoxanthum; D. ignipectus* | Anthreptes; n=18 | | 5.7 | |

| *Lonchura striata; L. punctulata* | Estrilda; n=16^#^ | 3.8×13+3.6+4.1=3.8 |
| --- | --- | --- |

*: Species numbers belong to the genus, the generation length is the same in all species in the same genus except for the genus Estrilda.

^#^: One species *Estrilda nigriloris* was data deficiency.

**References**

Dunning, J.B. (2007) CRC handbook of avian body masses, 2nd ed. Taylor and Francis, Boca Raton, FL.

del Hoyo, J., Elliott, A., Sargatal, J., Christie, D.A. & de Juana, E. (eds.) (2015). Handbook of the Birds of the World Alive. Lynx Edicions, Barcelona. (retrieved from http://www.hbw.com/ on 30 August 2015).

Zhao, Z. J. (2001). A handbook of the birds of China (2nd edition, in Chinese). Jilin Science Press.
